# Toward Optimized ^89^Zr-Immuno-PET: Side-by-Side Comparison of [^89^Zr]Zr-DFO-, [^89^Zr]Zr-3,4,3-(LI-1,2-HOPO)- and [^89^Zr]Zr-DFO*-Cetuximab for Tumor Imaging: Which Chelator Is the Most Suitable?

**DOI:** 10.3390/pharmaceutics14102114

**Published:** 2022-10-04

**Authors:** Helen Damerow, Xia Cheng, Valeska von Kiedrowski, Ralf Schirrmacher, Björn Wängler, Gert Fricker, Carmen Wängler

**Affiliations:** 1 Biomedical Chemistry, Clinic of Radiology and Nuclear Medicine, Medical Faculty Mannheim, Heidelberg University, 68167 Mannheim, Germany; 2 Molecular Imaging and Radiochemistry, Clinic of Radiology and Nuclear Medicine, Medical Faculty Mannheim, Heidelberg University, 68167 Mannheim, Germany; 3 Division of Oncologic Imaging, Department of Oncology, Faculty of Medicine and Dentistry, University of Alberta, Edmonton, AB T6G 1Z2, Canada; 4 Institute of Pharmacy and Molecular Biotechnology, University of Heidelberg, 69120 Heidelberg, Germany

**Keywords:** ^89^Zr, DFO, DFO*, 3,4,3-(LI-1,2-HOPO), bioconjugation, cetuximab, in vivo pharmacokinetics, kinetic inertness

## Abstract

^89^Zr represents a highly favorable positron emitter for application in immuno-PET (Positron Emission Tomography) imaging. Clinically, the ^89^Zr^4+^ ion is introduced into antibodies by complexation with desferrioxamine B. However, producing complexes of limited kinetic inertness. Therefore, several new chelators for ^89^Zr introduction have been developed over the last years. Of these, the direct comparison of the most relevant ones for clinical translation, DFO* and 3,4,3-(LI-1,2-HOPO), is still missing. Thus, we directly compared DFO with DFO* and 3,4,3-(LI-1,2-HOPO) immunoconjugates to identify the most suitable agent stable ^89^Zr-complexation. The chelators were introduced into cetuximab, and an optical analysis method was developed, enabling the efficient quantification of derivatization sites per protein. The cetuximab conjugates were efficiently obtained and radiolabeled with ^89^Zr at 37 °C within 30 min, giving the [^89^Zr]Zr-cetuximab derivatives in high radiochemical yields and purities of >99% as well as specific activities of 50 MBq/mg. The immunoreactive fraction of all ^89^Zr-labeled cetuximab derivatives was determined to be in the range of 86.5–88.1%. In vivo PET imaging and ex vivo biodistribution studies in tumor-bearing animals revealed a comparable and significantly higher kinetic inertness for both [^89^Zr]Zr-3,4,3-(LI-1,2-HOPO)-cetuximab and [^89^Zr]Zr-DFO*-cetuximab, compared to [^89^Zr]Zr-DFO-cetuximab. Of these, [^89^Zr]Zr-DFO*-cetuximab showed a considerably more favorable pharmacokinetic profile with significantly lower liver and spleen retention than [^89^Zr]Zr-3,4,3-(LI-1,2-HOPO)-cetuximab. Since [^89^Zr]Zr-DFO* demonstrates a very high kinetic inertness, paired with a highly favorable pharmacokinetic profile of the resulting antibody conjugate, DFO* currently represents the most suitable chelator candidate for stable ^89^Zr-radiolabeling of antibodies and clinical translation.

## 1. Introduction

Positron Emission Tomography (PET) is an important imaging modality for the visualization of malignancies in clinical routine. Compared to radiologic imaging techniques such as Computed Tomography (CT) and Magnetic Resonance Imaging (MRI), PET offers multiple advantages. It provides fully quantifiable imaging information and target-specific functional imaging of tumors on a molecular level. Due to this, PET does not only allow the identification of malignancies, but also their characterization in terms of metabolism, receptor status, vitality and aggressiveness. Hence, PET plays a key role in tumor delineation, staging, treatment planning and control, as well as assessment of therapy response and prognosis.

To be able to achieve this sensitive and target-specific imaging, bioactive carrier molecules are applied for target-specific delivery of the attached radioisotope being detected during the PET scan. For this purpose, different molecule classes can be used, among these small molecules, peptides and antibodies. Antibodies exhibit the advantage of being able to address their respective target structure with very high affinity and selectivity, usually resulting in a high target accumulation and high achievable tumor-to-background contrasts. However, antibodies display relatively slow pharmacokinetics, usually resulting in high image contrasts only several days after application. This is, however, not compatible with the short physical half-life of most clinically relevant PET radionuclides such as ^11^C, ^18^F, ^64^Cu and ^68^Ga, their half-lives being in the range of minutes to hours. ^89^Zr, which exhibits a significantly longer half-life of 3.27 days and has therefore established itself as the standard radionuclide for preclinical and clinical antibody-based immuno-PET imaging [1,2,3,4].

**Figure 1:**
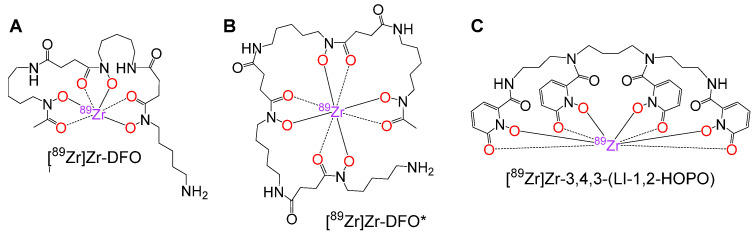
Structures of [^89^Zr]Zr-DFO (**A**), [^89^Zr]Zr-DFO* (**B**) and [^89^Zr]Zr-3,4,3-(LI-1,2-HOPO) (**C**).

For clinical application in immuno-PET imaging, ^89^Zr^4+^ is introduced into antibodies using the natural siderophore desferrioxamine B (DFO, Figure 1A), which, however, forms complexes of limited kinetic inertness with the radiometal under in vivo conditions [5,6]. The resulting release of ^89^Zr^4+^ from the complex is not only problematic due to a decreased image quality caused by increased background activity levels but also due to the deposition of the radiometal ion in mineral bone (due to attachment to hydroxyapatite), entailing a relevant dose to hematopoietic bone marrow [5,7] and potential masking of bone lesions [8].

Due to these challenges, considerable attempts were made within the last years to develop new chelating agents, being designed to completely saturate the coordination sphere of ^89^Zr^4+^ (requiring 8 instead of 6 coordinating atoms being offered by DFO) and to form kinetically inert complexes. Among these, some chelator candidates were identified, which showed a considerably improved kinetic inertness of the associated ^89^Zr-complexes, thus potentially lending themselves as suitable agents for clinical translation [5,6,7]. However, studies systematically comparing the properties of the developed chelating agents and respective ^89^Zr-complexes are scarce, leaving the question unanswered of which of the developed agents is the most suitable for clinical routine immuno-PET imaging applications.

Some recent complex challenge experiments and in vivo PET imaging studies on ^89^Zr-labeled antibodies in tumor-bearing mice compared relative stabilities of ^89^Zr-complexes with different chelators have been developed over the last years. These studies revealed some of these new chelating agents to be of high interest for further testing, and potential clinical translation, whereas several others could be excluded due to a relatively lower or similarly low kinetic inertness of their ^89^Zr-complexes compared to [^89^Zr]Zr-DFO [9,10,11,12,13,14,15,16].

Especially [^89^Zr]Zr-DFO* (Figure 1B) demonstrated a superior kinetic inertness compared to [^89^Zr]Zr-DFO and many other newly developed chelators [9,11,12,14]. Thus, DFO* seems to be the superior chelating agent for stable ^89^Zr-introduction into antibodies and ^89^Zr-based immuno-PET imaging. Besides DFO*, also the recently developed chelator 3,4,3-(LI-1,2-HOPO) has shown exceptional stability of its ^89^Zr-complex in immuno-PET of tumor-bearing animals [13] and thus represents a potential clinical alternative to DFO*.

Although DFO* has already been systematically tested in direct comparison against most other relevant chelating agents with respect to the kinetic inertness of the formed ^89^Zr-complexes, a side-by-side comparison of the kinetic inertness of [^89^Zr]Zr-DFO* and [^89^Zr]Zr-3,4,3-(LI-1,2-HOPO) (Figure 1C) under in vivo conditions and the influence of both complexes on the in vivo pharmacokinetics of respectively modified antibodies is still missing.

This side-by-side comparison between [^89^Zr]Zr-DFO* and [^89^Zr]Zr-3,4,3-(LI-1,2-HOPO) is the focus of the current work.

## 2. Materials and Methods

### 2.1. General

Chemicals and materials for in vitro assays. All chemicals were purchased from commercial sources in analytical grade quality and used without further purification unless otherwise stated. Water (HPLC grade) was obtained from Carl Roth (Karlsruhe, Germany), trifluoroacetic acid (TFA) (uvasol quality) from SigmaAldrich (Taufkirchen, Germany) and acetonitrile (MeCN) from Häberle Labortechnik (Lonsee-Ettlenschieß, Germany). H_2_O (Tracepur quality), hydrochloric acid (30%, suprapur quality) and sodium hydroxide (30%, suprapur quality) were purchased from Merck (Darmstadt, Deutschland). Bradford reagent was obtained from SigmaAldrich (Taufkirchen, Germany). (4-(1,2,4,5-Tetrazine-3-yl)phenyl)methanamine hydrochloride and 4-(1,2,4,5-tetrazine-3-yl)benzoic acid were obtained from Varimol (Stuttgart, Germany). (*E*)-cyclooct-4-en-NHS carbonate (TCO-NHS, **1**) and (*E*)-cyclooct-4-en-1-yl-(4-nitrophenyl) carbonate were purchased from SiChem (Bremen, Germany). Cetuximab (Erbitux) was purchased as a 5 mg/mL solution for infusion from Merck (Darmstadt, Germany) and Dulbecco’s phosphate-buffered saline (DPBS, 1X) was obtained from VWR (Bruchsal, Germany). HEPES (4-(2-hydroxyethyl)piperazine-1-ethanesulfonic acid) (ultrapure quality) from Gerbu Biotechnik GmbH (Heidelberg, Germany). All other chemicals and solvents were obtained from Carl Roth (Karlsruhe, Germany), Sigma Aldrich (Taufkirchen, Germany), TCI Deutschland GmbH (Eschborn, Germany) and Thermo Fisher GmbH (Kandel, Germany). The chelator tetrazines **4**–**6** were synthesized as described before [9]. [^89^Zr]Zr-oxalate solution in 1.0 M oxalic acid was purchased from PerkinElmer (NEZ308000MC, Rodgau, Germany). A431 human epidermoid carcinoma cells and HT-29 human colon adenocarcinoma cells were obtained from DSMZ (Braunschweig, Germany). McCoy’s 5A medium (1X, modified), Dulbecco’s Modified Eagle Medium (DMEM), Penicillin-Streptomycin (10,000 U/mL) as well as trypsin solution (0.25%), supplemented with ethylene diamine *tetra*-acetic acid (EDTA) and phenol red were purchased from Thermo Fisher GmbH (Kandel, Germany). Fetal calf serum (FCS) was obtained from Bio&Sell GmbH (Feucht, Deutschland). Instrumentation. HPLC (high-performance liquid chromatography): For HPLC chromatography, a Dionex UltiMate 3000 system from Thermo Fisher was used together with Chromeleon Software (Version 6.80). For analytical chromatography during chemical syntheses, a Chromolith Performance (RP-18e, 100–4.6 mm, Merck, Germany) and according to semipreparative purifications, Chromolith (RP-18e, 100–10 mm, Merck, Germany) columns were used, respectively. For chemical analytical and semipreparative HPLC, a flow rate of 4 mL/min and the eluents H_2_O and MeCN containing 0.1% TFA were used. For analysis of cetuximab, its bioconjugates and ^89^Zr-labeled analogs, the same HPLC system was used but the column was replaced by a size-exclusion chromatography (SEC) Superdex 200 Increase 10/300 GL column (10 × 300 mm, Cytiva, VWR, Bruchsal, Germany), being operated at a flow rate of 0.75 mL/min using phosphate buffer (0.05 M, pH 7.0), supplemented with NaCl (0.15 M) and NaN_3_ (0.01 M) as the eluent. For radio-analytical HPLC chromatography, the system was equipped with a radio detector GabiStar (Raytest, Straubenhardt, Germany). Sephadex gravity SEC columns (NAP-5, NAP-10 and NAP-25) were obtained from Cytiva and centrifugal Vivaspin 6 (30,000 MWCO) concentrators were purchased from Merck (Darmstadt, Germany). UV/VIS spectroscopy was performed on a BioSpectrometer kinetic from Eppendorf using 70 µL UV-cuvettes from Brand (Weinheim, Germany). Matrix-Assisted Laser Desorption/Ionization (MALDI) time-of-flight mass spectra were obtained utilizing a Bruker Daltonics Microflex spectrometer (Bruker Daltonics, Bremen, Germany). For high-resolution electrospray ionization mass spectroscopy (HR-ESI-MS), a Thermo Finnigan LTQ FT Ultra Fourier Transform Ion Cyclotron Resonance (Dreieich, Germany) mass spectrometer was used. Radioactivity measurements were performed using an ISOMED 2010 activimeter from NUVIATech Instruments (Dresden, Germany). γ-Counting was performed using a 2480 Wizard gamma counter system from Perkin Elmer and tissue activities were determined against standards taken from the injected tracer solutions. Radio-iTLC analyses were performed using iTLS-SG material (Agilent Technologies, Santa Clara, CA, USA) together with citrate buffer (0.1 M, pH 5) as the eluent and analyzed using a Scan-Ram Radio-TLC scanner from LabLogic (Koblenz, Germany) and the Laura software (version 4.1.12.89) of the same supplier. In vivo and ex vivo experiments. All animal experiments were performed in compliance with the German animal protection laws and protocols of the local committee (Regierungspräsidium Karlsruhe, approval number: 35-9185.81/G-266/17). For animal experiments, 4–5 weeks-old female nude mice (Balb/cAnN-Foxn1^nu/nu^/RJ, average weight 20 g) were purchased from Janvier (Le Genest-Saint-Isle, France). Static PET/CT imaging was performed over 60 minuntes on a triple-modality Bruker Albira II small-animal PET/CT/SPECT scanner (Karlsruhe, Germany). At the end of each PET scan, the CT scan was performed at 45 kVp, with currents of 0.4 mA (‘high dose, good resolution’) and 400 projections within 20 min. The PET images were reconstructed using the Albira Suite Reconstructor (Bruker) with a maximum likelihood estimation method (MLEM) algorithm with a Graphic Processing Unit (GPU). 12 iterations with a cubic voxel size of 0.5 mm as well as decay, scatter and random corrections were applied. CT images were reconstructed with 125 µm isotropic voxel size via filtered back projection (FBP). The reconstructed PET data were manually fused with the CT images using PMOD 3.6.0.8 and analyzed. Statistical analyses. Statistical comparisons of organ/tissue uptakes were performed by unpaired, two-tailed *t*-test using GraphPad Prism version 8.4.3.

### 2.2. Bradford Assay

First, a series of standards were prepared and analyzed to obtain values for a standard curve. For this purpose, a dilution series of cetuximab comprising 0, 0.25, 0.5, 0.75, 1, 1.25 mg/mL in DPBS buffer (0.1 M, pH 7.4) was prepared. For each dilution, 300 µL Bradford reagent was mixed with 10 µL of the respective sample. The mixture was gently mixed and transferred to 70 µL micro UV-cuvettes. After 20 min at ambient temperature, the absorbance at 595 nm was measured with a UV/VIS spectrometer. From these data, a standard curve was generated. The cetuximab samples being obtained during chemical modifications were treated and analyzed analogously to the samples of the dilution series, and their concentration was obtained by means of the standard curve equation.

### 2.3. Preparation of TCO-Cetuximab ***2***

The commercially available cetuximab solution was first re-buffered to a DPBS buffer containing Na_2_CO_3_, resulting in a pH of 8.6–8.8. A solution of **1** in DMSO (dimethyl sulfoxide, 4 equiv., 400 nmol, 0.106 mg, 2 mg/mL, 53.2 µL) was added to the cetuximab solution (100 nmol, 14.5 mg, 12 mg/mL, 1.2 mL). The reaction was incubated at ambient temperature for 2 h with gentle shaking. Afterwards, the purification was carried out using an SEC Sephadex NAP-25 column using DPBS (pH 7.4) as the eluent. **2** was obtained in a yield of 80% (4.3 mg/mL, 2.7 mL, 11.6 mg, 80 nmol).

### 2.4. Determination of the TCO-to-Cetuximab Ratio of ***2***

First, a series of standards of **3** was prepared and analyzed to obtain values for a standard curve. For this purpose, a dilution series comprising 0, 10, 25, 40, 250, 500, 750 and 1000 µg/mL of **3** in DPBS buffer (pH 7.4) was prepared. A sample of each solution was transferred to 70 µL micro-UV-cuvettes, and the absorbance at 515 nm was measured with a UV/VIS spectrometer. From these data, a standard curve was generated. The concentration of antibody-bound TCO in the solution of **2** (being obtained as described before) was determined by reacting a sample of the respective solution of **2** with an excess (10–15 equiv.) of **3**. A sample of the resulting solution was measured as described before at 515 nm and the concentration of **3** was determined by means of the standard curve equation. The amount of reacted **3** (quantity of applied **3**—the measured amount of **3** after reaction with **2**) equals the amount of TCO in the sample of **2**. As the cetuximab amount in the solution of **2** is known by the Bradford assay, the TCO-to-cetuximab ratio is obtained by dividing the amount of TCO by the amount of cetuximab.

### 2.5. Preparation of Chelator-Cetuximab-Bioconjugates ***7**–**9***

To a solution of TCO-cetuximab (**2**, 5 mg, 34.3 nmol) in DPBS buffer (pH 7.4, 1.15 mL) was added a solution of the respective chelator tetrazine **4**–**6** (8 equiv., 275 nmol) in DMSO (30 µL). The mixture was allowed to react for 5 h at ambient temperature with gentle shaking before the chelator-cetuximab-bioconjugates **7**–**9** were purified by SEC Sephadex NAP-10 or NAP-25 columns using DPBS (pH 7.4) as the eluent, giving **7**–**9** in a DPBS volume of 1.5–2.5 mL and yields of 88% (4.4 mg, 30.2 nmol).

### 2.6. ^89^Zr-Radiolabeling of ***7**–**9*** Yielding [^89^Zr]Zr-***7***–[^89^Zr]Zr-***9***

The chelator-cetuximab-bioconjugates **7**–**9** (1.1–1.4 mg, 7.5–9.6 nmol), obtained as described before, were re-buffered to HEPES buffer (0.25 M, pH 7.0, 220–280 µL) using size-exclusion ultrafiltration tubes with an MWCO (molecular weight cut-off) of 30,000 g/mol. To these solutions was added a solution of [^89^Zr]Zr-oxalate (0.1 M HCl solution, 55–70 MBq, ~80 µL) in HEPES buffer (0.25 M, pH 9.0, 100 µL), which was adjusted to pH 7.0–7.2 using NaOH solution (30%, 2–4 µL) so that a non-optimized specific activity of 50 MBq/mg cetuximab was obtained for all immunoconjugates. The respective reaction mixtures were incubated at 37 °C and the reaction process was monitored by radio-iTLC (Figures S1–S4). For all bioconjugates, an incorporation rate of [^89^Zr]Zr^4+^ of >99% was achieved after 30 min reaction time. The corresponding radio-HPLC analyses showed ^89^Zr-incorporation rates of >96%. Before further use in in vitro or in vivo experiments, the labeled products [^89^Zr]Zr-**7**–[^89^Zr]Zr-**9** were nevertheless again purified by SEC Sephadex NAP-5 columns as described before and obtained in recovery rates of 85–92% (without the following ultracentrifugation) and 81–87% (with the following ultracentrifugation) and radiochemical purities of >99%.

### 2.7. Cell Culture

Both cell lines, A431 and HT-29 cells, were grown at 37 °C in a humidified (>93% humidity) CO_2_ (5%) atmosphere and were split at 70–90% confluence. A431 epidermoid carcinoma cells were grown in DMEM, and HT-29 cells were grown in McCoy’s 5A (1X, modified) medium, both being supplemented with 10% (*v*/*v*) fetal bovine serum and 1% (*v*/*v*) penicillin-streptomycin (10,000 U/mL) and the medium was replaced every 2–3 days. For splitting, the medium was removed, and the cells were washed with DPBS and afterwards detached from the surface using a 0.25% trypsin solution for 5 min at 37 °C. After the enzymatic reaction was stopped by adding culture medium, the cells were seeded into new cell culture flasks. The medium was changed 24 h before each in vitro or in vivo experiment.

### 2.8. Determination of the Immunoreactive Fraction of [^89^Zr]Zr-***7***–[^89^Zr]Zr-***9***

For the determination of the immunoreactive fraction of [^89^Zr]Zr-**7**–[^89^Zr]Zr-**9**, a Lindmo assay was performed on A431 cells. For this purpose, the cells were transferred into low binding-Eppendorf tubes, suspended in DPBS + 1% BSA in a cell concentration of 6 × 10^6^ per 950 µL. 4 tubes were prepared for each experiment. To one of the tubes a solution of cetuximab (50 µg) in DPBS (pH 7.4, 50 µL) was added, resulting in a cetuximab concentration of 50 µg/mL. To the three other tubes, DPBS (pH 7.4, 50 µL) without cetuximab was added and all solutions were incubated on ice for 2 h. Of each cell suspension, a dilution series was prepared, comprising 3.0, 1.5, 0.75, 0.375 and 0.1875 × 10^6^ cells/mL. 500 µL of each cell suspension were taken for the following experiments and incubated with a solution of the respective labeled cetuximab derivative [^89^Zr]Zr-**7**, [^89^Zr]Zr-**8** or [^89^Zr]Zr-**9** (6 ng, 0.12 ng/µL, 50 µL, 0.15 kBq) in DPBS (pH 7.4) at 23 °C under gentle agitation for 2 h. After this time, the solution was separated from the cells by centrifugation (7000 rpm for 5 min) and transferred to gamma counter tubes. The remaining cell pellet was washed twice with DPBS (pH 7.4, 250 µL) and the washing solutions were added to the incubation solution obtained before. The washed cells were re-suspended in DPBS (750 µL, pH 7.4) and transferred to gamma counter tubes. Each experiment was performed three times, each being performed in triplicate.

### 2.9. In Vivo PET/CT Imaging and Ex Vivo Biodistribution of [^89^Zr]Zr-***7***–[^89^Zr]Zr-***9***

All animal experiments were performed in compliance with the German animal protection laws and protocols of the local committee (Regierungspräsidium Karlsruhe, approval number: 35-9185.81/G-266/17). For the animal studies, 4–5 weeks-old female nude Balb/c-AnN-Foxn-1^nu/nu^- Rj mice (Janvier, Le Genest-Saint-Isle, France) with an average weight of 20 g were used. For the inoculation of the HT-29 tumor, 2–5 × 10^6^ cells in DBPS buffer (pH 7.4, 100 µL) were injected subcutaneously into the right posterior flank under isoflurane anaesthesia. The tumor size was regularly measured with a calliper, and the examination by PET/CT was performed between day 12 to 14 after inoculation when the tumor reached an approximate diameter of 0.3–0.5 cm. Animals received 5.37 ± 2.38 MBq [^89^Zr]Zr-**7** (0.11 ± 0.05 mg cetuximab, *n* = 5), 5.73 ± 3.05 MBq [^89^Zr]Zr-**8** (0.11 ± 0.06 mg cetuximab, *n* = 7) or 4.42 ± 2.37 MBq [^89^Zr]Zr-**9** (0.09 ± 0.05 mg cetuximab, *n* = 7) by lateral tail vein injection, each cetuximab analog exhibiting a specific activity of 50 MBq/mg. Static PET/CT images were acquired directly after injection and also after 72 and 144 h p.i. After the end of the last scan, the animals were sacrificed at 145 h p.i. and all organs (tumor, blood, heart, lung, stomach, small intestine, cecum with large intestine, spleen, pancreas, liver, kidney, brain, muscle as well as the bones skull, sternum, right and left femur) were collected, weighed, and measured in a γ-counter. Three reference vials containing a known amount of ^89^Zr were also measured to calculate the injected dose per organ or tissue in kBq and determine the injected dose per gram organ or tissue (%ID/g).

## 3. Results and Discussion

### 3.1. Development of an Antibody Conjugation Approach Enabling the Exact Quantification of the Number of Derivatization Sites per Antibody

For the systematic comparison of the kinetic inertness of [^89^Zr]Zr-DFO* and [^89^Zr]Zr-3,4,3-(LI-1,2-HOPO) under in vivo conditions and the investigation of the influence of the respective chelating agent on the in vivo pharmacokinetics of modified biomolecules, both complexes have to be evaluated as part of antibody conjugates. An antibody conjugation of the ^89^Zr-complexes is mandatory to achieve their sufficiently long circulation in vivo. This is the prerequisite for potential complex challenge and release of [^89^Zr]Zr^4+^ from the respective complex as both chelating agents—DFO* and 3,4,3-(LI-1,2-HOPO)—were shown before to give ^89^Zr-complexes of high kinetic inertness under in vivo conditions. Further, the resulting ^89^Zr-labeled antibodies have to be systematically compared in an in vivo tumor model to assess their ability not only to stably incorporate and retain the radiometal but also to visualize the target structure and thus not to interfere with antigen binding or negatively influence in vivo pharmacokinetics.

For this purpose, cetuximab was chosen as full length antibody in the IgG format, efficiently targeting the epidermal growth factor receptor (EGFR) and being approved for the treatment of metastatic colorectal and head and neck cancer also showing a high efficacy for the treatment of non-small cell lung cancer [17]. To be able to directly compare the obtained in vivo pharmacokinetic data of [^89^Zr]Zr-DFO*-cetuximab and [^89^Zr]Zr-3,4,3-(LI-1,2-HOPO)-cetuximab, the chelating agents/complexes have to be introduced into the antibody using the same bioconjugation chemistry. Besides the number of derivatization sites per antibody molecule [18,19,20], also the type of chemical ligation can influence the obtained results [7]. In addition, [^89^Zr]Zr-DFO-cetuximab had to be synthesized under identical conditions, enabling a direct comparison of both promising alternatives (DFO* and 3,4,3-(LI-1,2-HOPO)) with the clinically applied standard chelating agent for ^89^Zr-labeling, DFO.

Ideally, the introduction of the chelator should be highly efficient under mild conditions, fully biocompatible, chemoselective and biorthogonal to enable efficient derivatization of antibody molecules. Further, it would be ideal to use a ligation technique that enables direct quantification of the number of introduced derivatization sites per antibody molecule as the latter determines the achievable immunoreactivity as well as the biodistribution of the bioconjugates [18,19,20]. One possibility to determine the number of chelators per antibody is by cumbersome isotopic dilution titration [21]. In most cases, the number of introduced derivatization sites is not quantified, and only the immunoreactivity of the conjugates is determined, which can entail a lengthy optimization process.

To address this issue, we developed a method for click chemistry-based chelator-antibody-modification relying on the inverse electron demand Diels-Alder (iEDDA) conjugation reaction between tetrazines and strained TCO (transcyclooctene) systems. This type of click chemistry reaction is very versatile and thus has found widespread use in radiochemistry [20,22,23]. In particular, this approach very recently showed that, in addition to conventional, very efficient antibody modification and subsequent direct one-step radiolabeling, it is also suitable for labeling antibodies in vivo via pretargeting [20].

Moreover, the iEDDA reaction enables the very simple, reliable and precise quantification of derivatization sites per antibody molecule, which is important to minimize negative effects on antibody immunoreactivity and in vivo pharmacokinetics by extensive derivatization.

**Figure 2:**
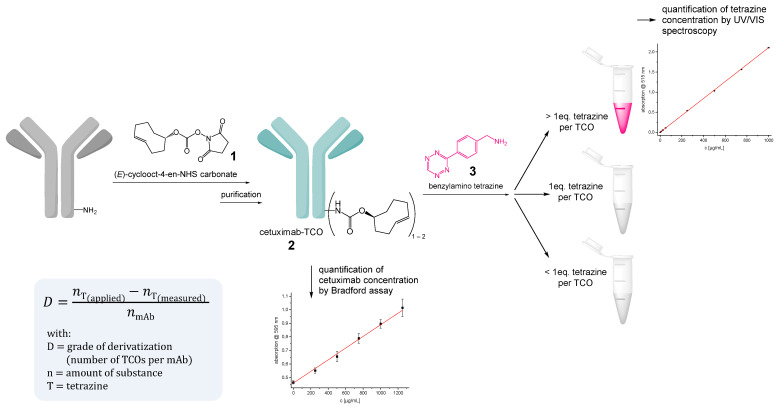
Schematic depiction of the antibody modification process with highly reactive TCO moieties, being followed by back-titration of TCO units and quantification using tetrazine and spectroscopic methods, giving the number of TCO modification sites per antibody molecule.

More precisely, a methodology was developed here by which the antibody is first reacted with a TCO-inducing agent and the number of introduced TCO units can then be efficiently and precisely quantified by back-titration with an intensely colored tetrazine (Figure 2). Following this approach, cetuximab was reacted with an excess of (*E*)-cyclooct-4-en-NHS carbonate (**1**) in an aqueous solution in a pH range of 8.6–8.8 for two hours at ambient temperature. An alternative attempt to use the respective *p*-nitrophenyl-active ester instead of the NHS ester gave lower conjugation efficiencies which furthermore considerably varied between batches. Thus, this route was not further followed. In contrast, the use of the NHS ester showed good TCO-conjugation efficiencies and resulted in highly reproducible results between batches (number of TCO molecules introduced per cetuximab molecule).

The purification of the cetuximab-TCO-conjugate (**2**) was performed by size-exclusion Sephadex columns using DPBS (Dulbecco’s Phosphate Buffered Saline solution, pH 7.4) as the eluent, giving the modified antibodies in 80–90% yield. An aliquot of the obtained pure product solution was first analyzed by Bradford assay to determine the precise protein concentration. Another aliquot of the solution was mixed with an excess of 10–15 equiv. of (4-(1,2,4,5-tetrazin-3-yl)phenyl)methanamine (**3**) and the amount of unreacted and thus excess tetrazine was determined by UV/VIS spectroscopy using a previously measured standard curve. By subtracting unreacted from applied tetrazine, the amount of TCO in the solution was obtained. Together with the previously determined amount of antibody protein, the number of TCO molecules per antibody is obtained. This approach implies, of course, that the iEDDA reaction between TCO and tetrazine is quantitative, which we were able to confirm prior to the development of the process by reacting a series of different and also equimolar known amounts of TCO and tetrazine and monitoring the amount of residual colored tetrazine by UV/VIS spectroscopy, with the measured values matching the expected ones.

The developed method allows the number of derivatization sites per antibody molecule to be determined very rapidly, simply and with great accuracy, which is a considerable advantage over conventional methods for quantification of antibody-chelator-modification sites.

We aimed to introduce an overall number of 1–2 derivatization sites per antibody molecule to limit the influence of chemical modifications on immunoreactivity and biodistribution of our radioligands (vide supra). Different amounts of **1** were applied for cetuximab modification and the number of derivatization sites was determined. The results of these experiments can be found in Table 1 and prompted us to use an excess of 4 equiv. (*E*)-cyclooct-4-en-NHS carbonate for cetuximab-TCO-modification, yielding the targeted derivatization.

### 3.2. Preparation of Cetuximab-Chelator-Conjugates ***7**–**9***

**Figure 3:**
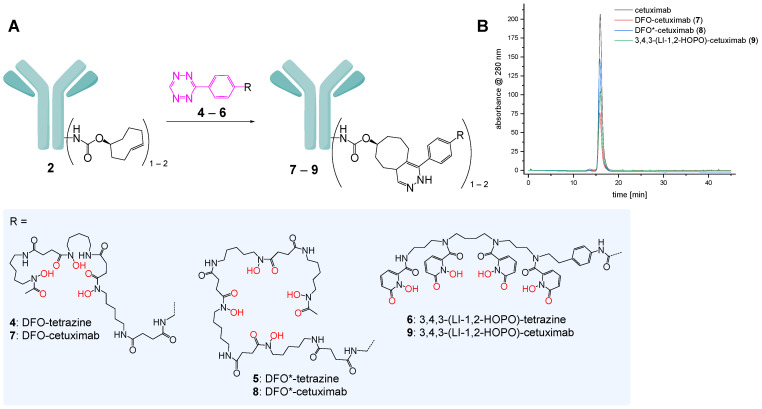
Schematic depiction of the preparation of the chelator-cetuximab-bioconjugates **7**–**9** from cetuximab-TCO **2** and the chelator-tetrazines **4**–**6** (**A**) and size-exclusion HPLC analyses of cetuximab and its chelator-modified analogs **7**–**9** (**B**).

For the subsequent modification of cetuximab-TCO **2** with DFO, DFO* and 3,4,3-(LI-1,2-HOPO) by iEDDA, being highly efficient at ambient temperature and physiological pH [22,23], tetrazine-modified analogs of the chelators (**4**–**6**, Figure 3A) were prepared as described before [9] and applied in the following cetuximab bioconjugation reactions. So far, these chelator tetrazines were only used for conjugation to one small peptide, the subsequent ^89^Zr-radiolabeling of the conjugates and comparative testing of the relative kinetic inertnesses of the complexes against challenge, ^89^Zr^4+^ liberation and transchelation. However, neither a conjugation to an antibody and ^89^Zr-labeling of the conjugates nor an in vivo application of the ^89^Zr-labeled agents alone or as bioconjugates has been disclosed yet. Nevertheless, both chelators **5** and **6** showed an extraordinarily high inertness of the formed ^89^Zr-complexes in this previous study [9], rendering them equally suited for in vivo application.

For the preparation of the chelator-cetuximab-bioconjugates **7**–**9**, a freshly prepared solution of **2** was reacted with an 8-fold excess of the respective chelator tetrazine for five hours before the chelator-modified antibodies were purified and concentrated using size-exclusion chromatography and ultrafiltration (MWCO of 30,000 g/mol). Also after this modification step, the protein content was quantified by Bradford assay and indicated recovery rates of 80–89% after chelator conjugation and purification. Size-exclusion HPLC analyses of the obtained products demonstrated the bioconjugates to be pure, showing neither residual **4**–**6** and only a very low ratio of aggregated protein (Figure 3B).

### 3.3. ^89^Zr-Radiolabeling of Cetuximab-Chelator-Conjugates ***7**–**9***

**Figure 4:**
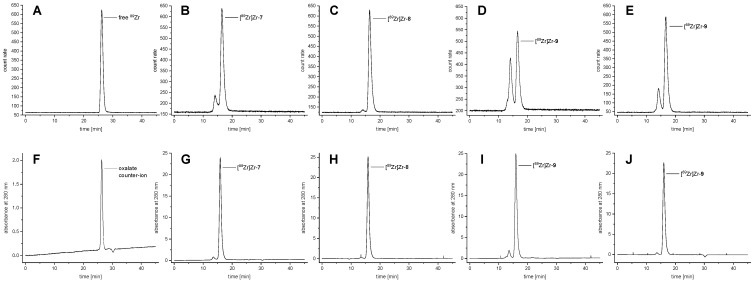
Analytical size-exclusion radio-HPLC chromatograms of [^89^Zr]Zr-oxalate (as reference) and the labeled antibodies [^89^Zr]Zr-**7**–[^89^Zr]Zr-**9**. Radio-traces are depicted in panel (**A**–**E**) and corresponding UV traces in panels (**F**–**J**). Depicted are chromatograms of [^89^Zr]Zr-oxalate (**A**,**F**), [^89^Zr]Zr-**7** (**B**,**G**), [^89^Zr]Zr-**8** (**C**,**H**), [^89^Zr]Zr-**9**, labeled with 50 MBq/mg protein (**D**,**I**) and [^89^Zr]Zr-**9**, labeled with 25 MBq/mg protein (**E**,**J**).

^89^Zr-radiolabeling of the chelator-cetuximab-bioconjugates **7**–**9** was performed in HEPES buffer (HEPES = 4-(2-Hydroxyethyl)piperazine-1-ethanesulfonic acid) by incubating 1.1–1.4 mg (7.5–9.6 nmol) of **7**–**9** with 55–70 MBq [^89^Zr]Zr-oxalate solution at mild reaction conditions of 37 °C and pH 7.0–7.2 within 30 min. Within this time, all reactions were quantitative (>99%) as determined by analytical size-exclusion radio-HPLC (Figure 4) and radio-iTLC (Figures S1–S4), giving the products [^89^Zr]Zr-**7**–[^89^Zr]Zr-**9** in non-optimized specific and molar activities of 50 MBq/mg and 7.3 GBq/µmol, respectively, being in the typical range for ^89^Zr-labeled mAbs [14,24].

Preceding further in vitro and in vivo evaluations of the agents, they were again purified by size-exclusion chromatography and for in vivo application, the solutions were further concentrated using ultracentrifugation with a MWCO of 30,000 g/mol.

Despite the very efficient ^89^Zr-incorporation into the modified cetuximab derivatives **7**–**9**, clear differences were observed between the antibody labeling reactions, depending on the respective chelator used.

As is clearly visible in the analytical size-exclusion radio-HPLC chromatograms (Figure 4B–E), the ^89^Zr-labeling of the chelator-cetuximab bioconjugates **7**–**9** produced, in addition to the expected product (peak at 16.5 min), another ^89^Zr-labeled species (peak at 14.1 min), which can be assigned to a dimeric cetuximab aggregate, an effect having been described before for ^89^Zr-labeled trastuzumab DFO-, DFO-cyclo*- and DFO*-conjugates as well [14]. This effect was found least pronounced for [^89^Zr]Zr-DFO*-cetuximab ([^89^Zr]Zr-**8**, Figure 4C) and is also negligible for [^89^Zr]Zr-DFO-cetuximab ([^89^Zr]Zr-**7**, Figure 4B). In contrast, labeling of **9** under identical conditions, giving [^89^Zr]Zr-3,4,3-(LI-1,2-HOPO)-cetuximab ([^89^Zr]Zr-**9**), showed an explicit dimer aggregate formation (Figure 4D), which was dependent on the amount of [^89^Zr]Zr^4+^ used; If the amount of [^89^Zr]Zr^4+^ and thus the resulting specific activity was decreased by half, the formation of the dimer was significantly reduced (Figure 4E vs. Figure 4D). Interestingly, this pronounced aggregate formation in the case of the 3,4,3-(LI-1,2-HOPO)-mAb-conjugates could also be detected in the respective UV chromatograms (Figure 4I,J) although to a much lower extent as compared to the radio-traces (Figure 4D vs. Figure 4I and Figure 4E vs. Figure 4J), leading to the assumption that the [^89^Zr]Zr^4+^ itself or another component of the ^89^Zr-stock solution might induce or at least supports the observed aggregate formation although the mechanism for this process is not clear. Since all ^89^Zr-radiolabeling reactions were performed under identical conditions using identically prepared chelator-cetuximab-conjugates and the observation of aggregate formation is negligible in case of [^89^Zr]Zr-DFO-cetuximab ([^89^Zr]Zr-**7**) and [^89^Zr]Zr-DFO*-cetuximab ([^89^Zr]Zr-**8**), it can be assumed that the effect of dimer aggregate formation is related to the chelating agent 3,4,3-(LI-1,2-HOPO) itself. This explanation would also be consistent with data from other studies observing that hydrophobic molecules introduced into the antibody can promote aggregate appearance in antibody-drug-conjugates [25]. The formation of different conformers of the [^89^Zr]Zr-3,4,3-(LI-1,2-HOPO)-complex, as being reported in a recent study on the ^89^Zr-labeled 3,4,3-(LI-1,2-HOPO)-c(RGDfK) peptide [9], was not observed in our present study—as expectable due to the size of the labeled antibody biomolecule, necessitating the analysis of the ^89^Zr-labeled IgGs to be performed by size-exclusion HPLC instead of standard C18 material-based HPLC chromatography used for small molecules.

### 3.4. Determination of the Immunoreactive Fraction of [^89^Zr]Zr-***7***–[^89^Zr]Zr-***9***

In the following, the immunoreactivity of the radiolabeled chelator-cetuximab-bioconjugates [^89^Zr]Zr-**7**–[^89^Zr]Zr-**9** was determined by Lindmo assay [26] to investigate potential influences of the respectively used chelating agent on the biological activity of the mAb molecule. Further, we intended to rule out a negative effect on immunoreactivity by the formation of the cetuximab aggregate in case of [^89^Zr]Zr-3,4,3-(LI-1,2-HOPO)-cetuximab ([^89^Zr]Zr-**9**) (vide supra).

**Figure 5:**
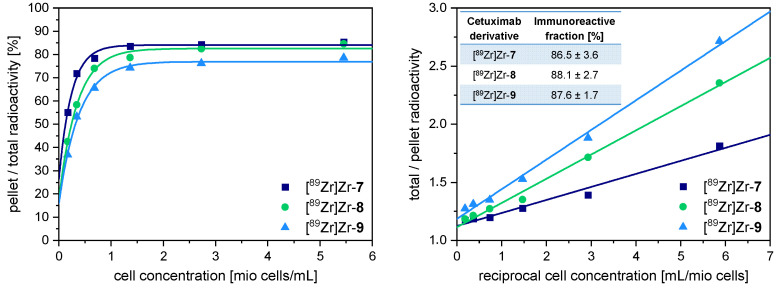
Results of the determination of the immunoreactive fraction of [^89^Zr]Zr-**7**–[^89^Zr]Zr-**9** on A431 cells. Shown are exemplary Lindmo plots as obtained during each individual experiment; immunoreactive fractions were obtained from three independent experiments, each performed in triplicate.

For this purpose, human epidermoid carcinoma A431 cells, highly overexpressing the target EGFR [27] and thus being the standard cell line for evaluation of EGFR-specific radiotracers [28], were used. Briefly, 0.15 kBq [^89^Zr]Zr-**7**, [^89^Zr]Zr-**8** or [^89^Zr]Zr-**9** were added to different concentrations of A431 cells in DPBS. The cells were separated from the solution by centrifugation and the cell-bound activity was determined as well as the total activity applied. Blocking experiments using an excess of unlabeled cetuximab confirmed unspecific binding to be below 2% for every dilution tested. The immunoreactivity of each labeled antibody and every individual experiment was determined by a Lindmo plot (Figure 5). For all chelator-cetuximab- bioconjugates, highly preserved immunoreactive fractions of 86.5 ± 3.6%, 88.1 ± 2.7% and 87.6 ± 1.7% were obtained for [^89^Zr]Zr-**7**, [^89^Zr]Zr-**8** and [^89^Zr]Zr-**9**, respectively.

Thus, neither the formation of non-covalent cetuximab aggregates nor the respective chelating agent used showed any significant influence on the immunoreactivity of the correspondingly modified antibodies. Judging from the in vitro binding affinity data only, all ^89^Zr-cetuximab derivatives [^89^Zr]Zr-**7**–[^89^Zr]Zr-**9** appear equally suited for the application in immuno-PET imaging studies of EGFR-positive tumors.

### 3.5. Immuno-PET/CT Imaging of [^89^Zr]Zr-***7***–[^89^Zr]Zr-***9***

The previously prepared radio-immunoconjugates [^89^Zr]Zr-**7**–[^89^Zr]Zr-**9** were investigated in direct comparison under identical conditions in an HT-29 tumor-bearing xenograft mouse model with respect to in vivo pharmacokinetics and ex vivo biodistribution properties.

**Figure 6:**
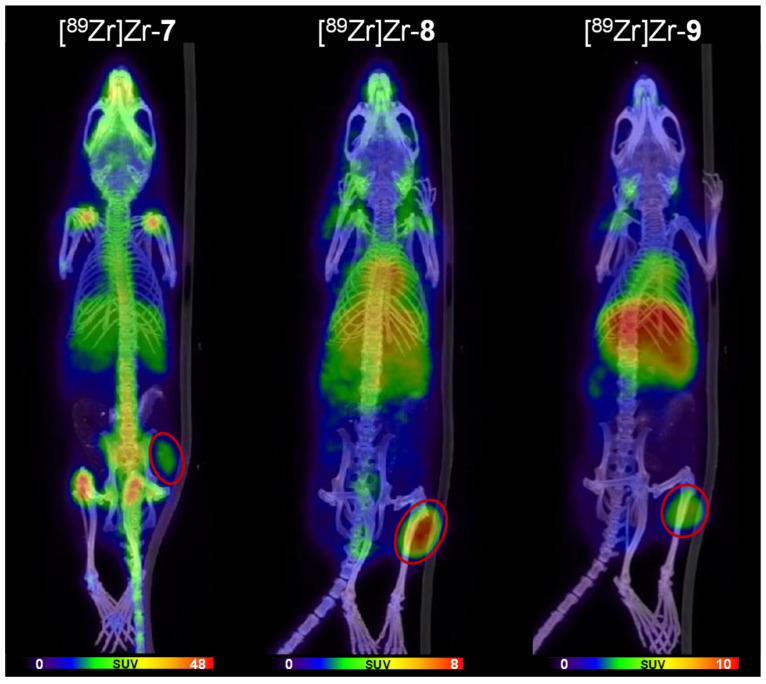
Representative small animal positron emission tomography/computed tomography (PET/CT) images obtained for [^89^Zr]Zr-DFO-cetuximab ([^89^Zr]Zr-**7**), [^89^Zr]Zr-DFO*-cetuximab ([^89^Zr]Zr-**8**) and [^89^Zr]Zr-3,4,3-(LI-1,2-HOPO)-cetuximab ([^89^Zr]Zr-**9**) in HT-29 tumor-bearing xenograft nude mice. The images show maximum intensity projections (MIPs) of the whole animals at 144 h post injection and the tumors are encircled.

Human colorectal cancer HT-29 cells, like A431 cells, overexpress EGFR to a very high extent and thus represent, in the form of xenograft mouse models, a standard in vivo system for the evaluation of radiolabeled EGFR-specific antibodies [29]. The in vivo imaging and ex vivo studies were performed in HT-29 tumor-bearing female Balb/cAnN-Foxn1^nu/nu^/RJ nude mice and the tumors were induced by subcutaneous tumor cell inoculation into the right flank of the animals. The animals received the respective radiotracer via lateral tail vein injection (5.37 ± 2.38 MBq of [^89^Zr]Zr-**7**, 5.73 ± 3.05 MBq of [^89^Zr]Zr-**8** or 4.42 ± 2.37 MBq [^89^Zr]Zr-**9**) and PET/CT scans were performed directly after intravenous injection, at 72 h p.i. and 144 h p.i. (p.i. = post injection). The results of the PET/CT scans are depicted in Figure 6 and Figure S5.

As can be expected, due to the slow pharmacokinetic profile of full-length antibodies in the IgG format, all ^89^Zr-labeled cetuximab derivatives showed only negligible tumor uptakes within the first hour p.i. and a rather moderate tumor visualization ability at 72 h p.i. (Figure S5). In contrast, a clear tumor visualization is possible with all agents at 144 h p.i. (Figure 6).

**Figure 7:**
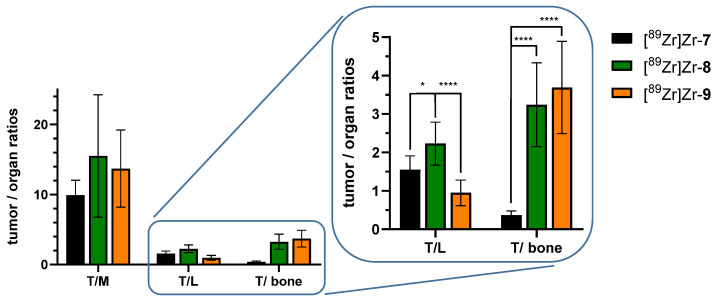
Tumor-to-organ ratios for the most relevant organs (tumor/muscle, tumor/liver and tumor/bone (femur)) as determined by ex vivo biodistribution studies in HT-29 tumor-bearing xenograft nude mice performed at 145 h p.i. using [^89^Zr]Zr-**7** (5.37 ± 2.38 MBq, *n* = 5), [^89^Zr]Zr-**8** (5.73 ± 3.05 MBq, *n* = 7) and [^89^Zr]Zr-**9** (4.42 ± 2.37 MBq, *n* = 7). *: *p* < 0.05, ****: *p* < 0.0001.

However, considerable differences in biodistribution can be observed from the PET/CT images after this time between the agents. Of all bioconjugates, the DFO-cetuximab derivative [^89^Zr]Zr-**7** showed the fastest clearance with low residual activity amounts in the blood and the excretory organs and also the by far the highest uptake of [^89^Zr]Zr^4+^ in mineral bone, being reflected in a strong accumulation in hip and shoulder joints, elbows, knees and vertebral bodies at 144 h p.i. (Figure 6, left panel and Figure 7). In detail, the highest uptakes in mineral bone were detected for the femurs (up to 19.68 ± 4.35 %ID/g), followed by 11.66 ± 0.14 %ID/g for the skull bone and 5.56 ± 2.33 %ID/g for the sternum (Table 2). That the uptake into mineral bone varies between different bones studied has been described before [30] and also strongly varying uptake values from below 10% ID/g bone tissue [30,31] to up to 15% [14,21] or even higher [32] have been described. Thus, the relatively high bone radioactivity uptake in the case of application of [^89^Zr]Zr-**7** is in line with former reports.

The high radioactivity uptake in mineral bone in the case of the DFO-cetuximab derivative [^89^Zr]Zr-**7** further matches the fast clearance of the activity from circulation and its comparatively lower tumor uptake compared to [^89^Zr]Zr-**8** and [^89^Zr]Zr-**9** (*p* < 0.05). This indicates a fast challenge of the [^89^Zr]Zr-DFO-complex under physiological conditions, resulting in the liberation of the radiometal cation and its deposition in the hydroxyapatite of the bones. Together, these results impressively confirm the results of many preceding studies showing the low kinetic inertness of the [^89^Zr]Zr-DFO-complex [2].

In contrast, both the DFO*- and 3,4,3-(LI-1,2-HOPO)-cetuximab derivatives [^89^Zr]Zr-**8** and [^89^Zr]Zr-**9** showed no relevant radiometal deposits in mineral bone in the PET images (Figure 6, middle and right panel), demonstrating a significantly higher kinetic inertness of the [^89^Zr]Zr-DFO*- and [^89^Zr]Zr-3,4,3-(LI-1,2-HOPO)-complexes compared to [^89^Zr]Zr-DFO (*p* < 0.0001).

Although the ^89^Zr-complexes of both [^89^Zr]Zr-**8** and [^89^Zr]Zr-**9** demonstrated a much higher kinetic inertness compared to [^89^Zr]Zr-**7**, DFO* and 3,4,3-(LI-1,2-HOPO) are not equally well suited for in vivo imaging purposes as [^89^Zr]Zr-**8** and [^89^Zr]Zr-**9** differed markedly in their pharmacokinetic profile regarding accumulation in liver and spleen: [^89^Zr]Zr-**9** demonstrated a significantly higher uptake in liver (*p* < 0.0001) and spleen (*p* < 0.02) compared to [^89^Zr]Zr-**8** at 145 h p.i., also being clearly visible in the PET/CT images as early as directly after injection (Figure S5) but also during the later scans (Figure S5 and Figure 6). This visual impression is confirmed by in the ex vivo biodistribution experiments being performed directly after the last PET/CT scan at 145 h p.i. (Figure 7 and Table 2).

In contrast, activity accumulation did not significantly differ between both agents [^89^Zr]Zr-**8** and [^89^Zr]Zr-**9** in blood and the other organs and tissues (muscle, brain, heart, lung, pancreas, kidneys, stomach, intestines and mineral bone, Table 2).

Together, these results indicate the high kinetic inertness of both, the [^89^Zr]Zr-DFO*- and [^89^Zr]Zr-3,4,3-(LI-1,2-HOPO)-complexes under in vivo conditions, confirming the results of our very recent comparative study on the relative stability and inertness of [^89^Zr]Zr-DFO-, [^89^Zr]Zr-DFO*-, [^89^Zr]Zr-3,4,3-(LI-1,2-HOPO)- and [^89^Zr]Zr-CTH-36-complexes [9].

However, if the remaining organ distribution is also taken into account, there is a clear difference between the two agents, since 3,4,3-(LI-1,2-HOPO) demonstrates an increased uptake of its immunoconjugate [^89^Zr]Zr-**9** in liver and spleen. Although [^89^Zr]Zr-3,4,3-(LI-1,2-HOPO)-labeled trastuzumab demonstrated no such higher liver and spleen uptake compared to its DFO-counterpart [^89^Zr]Zr-DFO-trastuzumab in a former study [13], it seems likely that the higher uptake of the 3,4,3-(LI-1,2-HOPO)-cetuximab derivative [^89^Zr]Zr-**9** observed here is a result of the higher lipophilicity of 3,4,3-(LI-1,2-HOPO) compared to DFO and DFO* [9]. That even a small lipophilic molecule introduced into an IgG molecule can result in a significantly increased accumulation in the mentioned organs has already been shown before [33]. Alternatively, the higher uptake of [^89^Zr]Zr-**9** in liver and spleen compared to [^89^Zr]Zr-**8** could also be a result of the observed aggregate formation in case of [^89^Zr]Zr-**9** (vide supra), being, however, also directly connected to the use of 3,4,3-(LI-1,2-HOPO) as this effect has not been observed in case of the application of DFO* for antibody modification and subsequent ^89^Zr-labeling.

This unspecific uptake of [^89^Zr]Zr-3,4,3-(LI-1,2-HOPO)-cetuximab is unfavorable as it could impede the detection and delineation of liver lesions (e.g., in hepatocellular carcinoma and liver metastases [34,35,36]) and thus influences the choice of the most appropriate chelating agent for stable ^89^Zr-antibody-labeling in favor of DFO* when using the introduced bioconjugation approach.

Based on the results obtained, it can be concluded that although DFO* and 3,4,3-(LI-1,2-HOPO) form ^89^Zr-complexes with comparably high kinetic inertness, the use of 3,4,3-(LI-1,2-HOPO) results in less optimal pharmacokinetics of the resulting ^89^Zr-labeled antibody molecule. Therefore, DFO* currently represents the most suitable candidate for stable ^89^Zr-radiolabeling of biomolecules and, thus clinical translation to replace DFO, the current clinical standard for ^89^Zr-labeling and immuno-PET imaging.

## 4. Conclusions

We were able to show that DFO* as well as 3,4,3-(LI-1,2-HOPO) form ^89^Zr-complexes of high kinetic inertness under in vivo immuno-PET imaging conditions even after one week post injection. In stark contrast to the respective DFO-complex, a significantly reduced release of the radiometal was observed, resulting in a low activity accumulation in mineral bone and high achievable tumor-to-background ratios in immuno-PET imaging.

However, [^89^Zr]Zr-3,4,3-(LI-1,2-HOPO)-cetuximab, besides showing high ^89^Zr-complex inertness, displayed a significantly higher uptake in liver and spleen compared to [^89^Zr]Zr-DFO*-cetuximab, resulting in lower tumor-to-background-ratios for these organs. No differences were observed between both ^89^Zr-cetuximab derivatives for other organs and tissues.

In conclusion, DFO* represents the most suitable chelator candidate for stable ^89^Zr-radiolabeling of antibodies. Hence, its clinical translation should be facilitated to replace DFO as the clinical standard for ^89^Zr-labeling and immuno-PET imaging.

## Figures and Tables

**Table 1:** Summary of the results of the systematic investigation of the derivatization efficiency of cetuximab using different excesses of (*E*)-cyclooct-4-en-NHS carbonate (**1**).

Excess of 1 per Cetuximab [equiv.]	Resulting TCO/Cetuximab Ratio
20	5.4 ± 0.3 (*n* = 3)
7	2.8 ± 0.5 (*n* = 3)
4	1.4 ± 0.3 (*n* = 6)
3	0.9 ± 0.3 (*n* = 2)

**Table 2:** Results of the ex vivo biodistribution studies obtained at 145 h p.i. for [^89^Zr]Zr-**7** (5.37 ± 2.38 MBq, *n* = 5), [^89^Zr]Zr-**8** (5.73 ± 3.05 MBq, *n* = 7) and [^89^Zr]Zr-**9** (4.42 ± 2.37 MBq, *n* = 7) in HT-29 tumor-bearing xenograft nude mice, expressed as %ID/g. The values are given as mean ± standard deviation.

Organ	[^89^Zr]Zr-7	[^89^Zr]Zr-8	[^89^Zr]Zr-9
tumor	7.23 ± 1.52	9.15 ± 1.88	9.31 ± 2.04
blood	4.95 ± 0.39	11.31 ± 1.23	8.83 ± 1.45
muscle	0.73 ± 0.04	0.59 ± 0.31	0.68 ± 0.23
brain	0.19 ± 0.05	0.27 ± 0.07	0.20 ± 0.04
heart	2.10 ± 0.41	2.73 ± 1.12	2.56 ± 1.20
lung	1.72 ± 1.42	4.91 ± 1.14	4.14 ± 1.02
spleen	3.29 ± 0.13	3.57 ± 1.02	5.54 ± 1.22
pancreas	0.42 ± 0.14	0.72 ± 0.17	0.61 ± 0.17
kidney	2.87 ± 0.16	4.08 ± 0.70	4.04 ± 0.95
liver	4.66 ± 0.49	4.11 ± 0.61	9.80 ± 2.58
stomach	0.74 ± 0.22	0.83 ± 0.18	1.07 ± 0.30
small intestine	0.84 ± 0.05	1.10 ± 0.34	1.31 ± 0.20
large intestine	0.51 ± 0.21	0.78 ± 0.21	0.82 ± 0.17
right femur	19.68 ± 4.35	2.82 ± 0.75	2.52 ± 0.60
left femur	18.62 ± 5.06	2.81 ± 0.96	2.64 ± 0.25
sternum	5.56 ± 2.33	2.82 ± 0.80	2.81 ± 0.39
skull	11.66 ± 0.14	2.76 ± 0.84	2.64 ± 0.25

## Data Availability

All relevant data is available in the manuscript or the supporting information.

## Supplementary Materials

The following supporting information can be downloaded at: https://www.mdpi.com/article/10.3390/pharmaceutics14102114/s1; Radio-iTLC analyses of [^89^Zr]Zr-**7**, [^89^Zr]Zr-**8** and [^89^Zr]Zr-**9** (Figures S1–S4) as well as in vivo PET/CT images for [^89^Zr]Zr-**7**, [^89^Zr]Zr-**8** and [^89^Zr]Zr-**9** at 0 h, 72 h and 144 h p.i. (Figure S5) and SUV_bw_ values obtained from the PET scans for selected organs and tissues (Table S1).
