# Supplementary material for: Toward Optimized 89Zr-Immuno-PET: Side-by-Side Comparison of [89Zr]Zr-DFO-, [89Zr]Zr-3,4,3-(LI-1,2-HOPO)- and [89Zr]Zr-DFO*-Cetuximab for Tumor Imaging: Which Chelator Is the Most Suitable?
Source: Pharmaceutics. 2022 Oct 4;14(10):2114. doi: 10.3390/pharmaceutics14102114 (PMC9611803; doi:10.3390/pharmaceutics14102114)
Supplement: Supplementary file 1 [file pharmaceutics-14-02114-s001.zip › pharmaceutics-1947242-supplementary-done.pdf]

pharmaceutics

Supplementary Materials: Toward Optimized ^{89}Zr-Immuno-PET: Side-by-Side Comparison of [^{89}Zr]Zr-DFO-, [^{89}Zr]Zr-3,4,3-(LI-1,2-HOPO)- and [^{89}Zr]Zr-DFO*-Cetuximab for Tumor Imaging—Which Chelator is the Most Suitable?

Helen Damerow, Xia Cheng, Valeska von Kiedrowski, Ralf Schirrmacher, Björn Wängler, Gert Fricker and Carmen Wängler

Content

Radio-iTLC analyses of [^{89}Zr]Zr-**7**, [^{89}Zr]Zr-**8** and [^{89}Zr]Zr-**9** before and after purification as well as free [^{89}Zr]Zr^{4+} as reference (Fig. S1 – S4) page 2

In vivo PET/CT images of [^{89}Zr]Zr-**7**, [^{89}Zr]Zr-**8** and [^{89}Zr]Zr-**9** at 0h, 72h and 144h p.i. (Fig. S5) page 4

SUV_{bw} values for selected organs and tissues obtained by PET imaging page 5

Radio-iTLC analyses of [^{89}Zr]Zr-**7**, [^{89}Zr]Zr-**8** and [^{89}Zr]Zr-**9** before and after purification as well as free [^{89}Zr]Zr-oxalate as reference. All radio-iTLC analyses were performed using iTLS-SG material with citrate buffer (0.1 M, pH 5) as the eluent and developed under identical conditions.

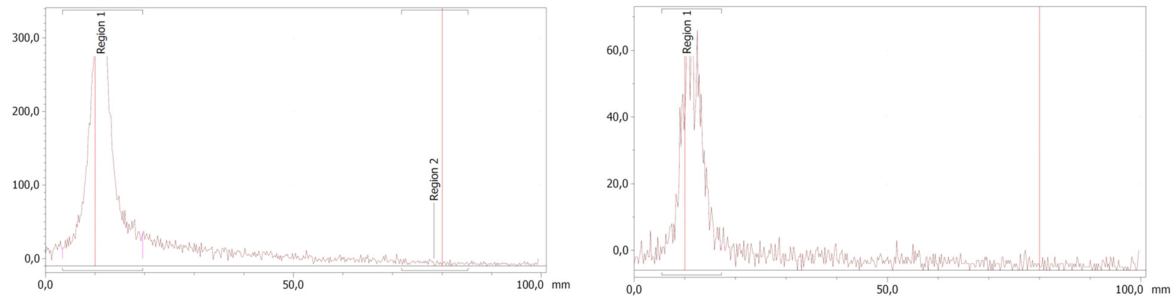

Figure S1: Radio-iTLC analysis of [^{89}Zr]Zr-**7** before (left) and after (right) purification by size-exclusion chromatography.

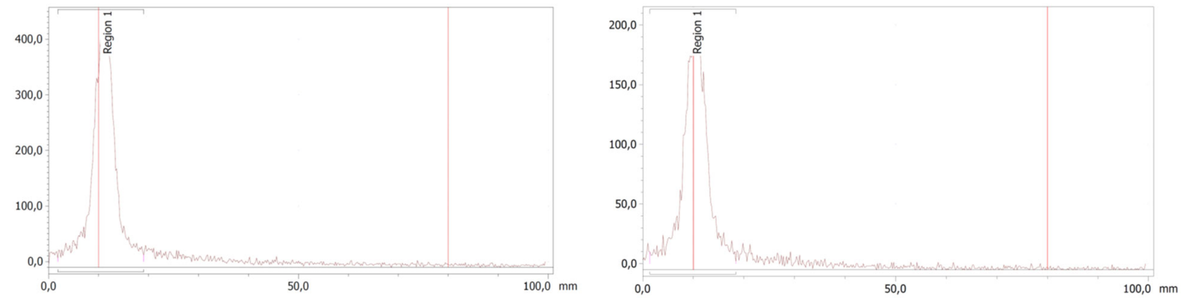

Figure S2: Radio-iTLC analysis of [^{89}Zr]Zr-**8** before (left) and after (right) purification by size-exclusion chromatography.

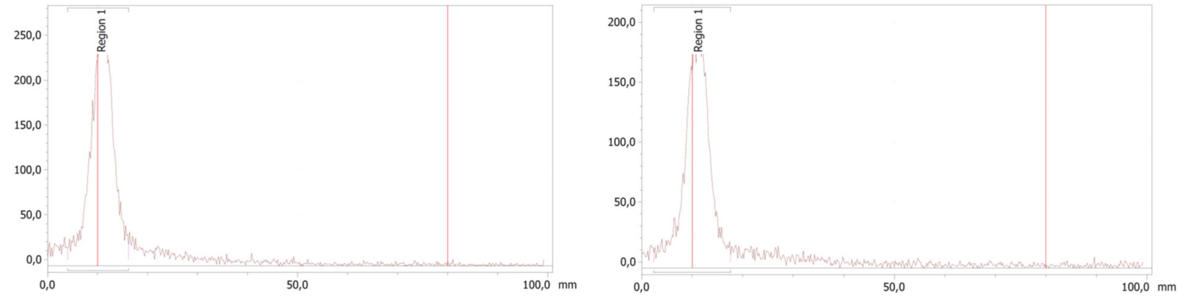

Figure S3: Radio-iTLC analysis of [^{89}Zr]Zr-**9** before (left) and after (right) purification by size-exclusion chromatography.

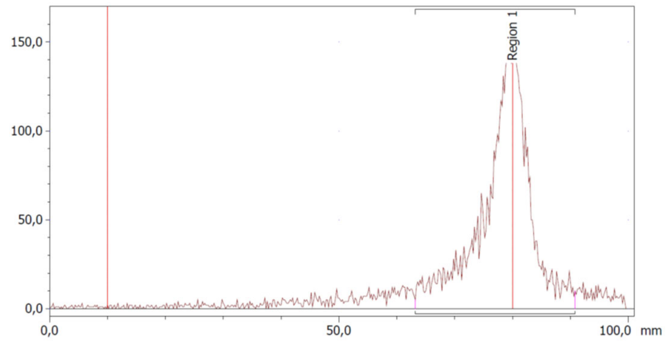

Figure S4: Radio-iTLC analysis of [^{89}Zr]Zr-oxalate.

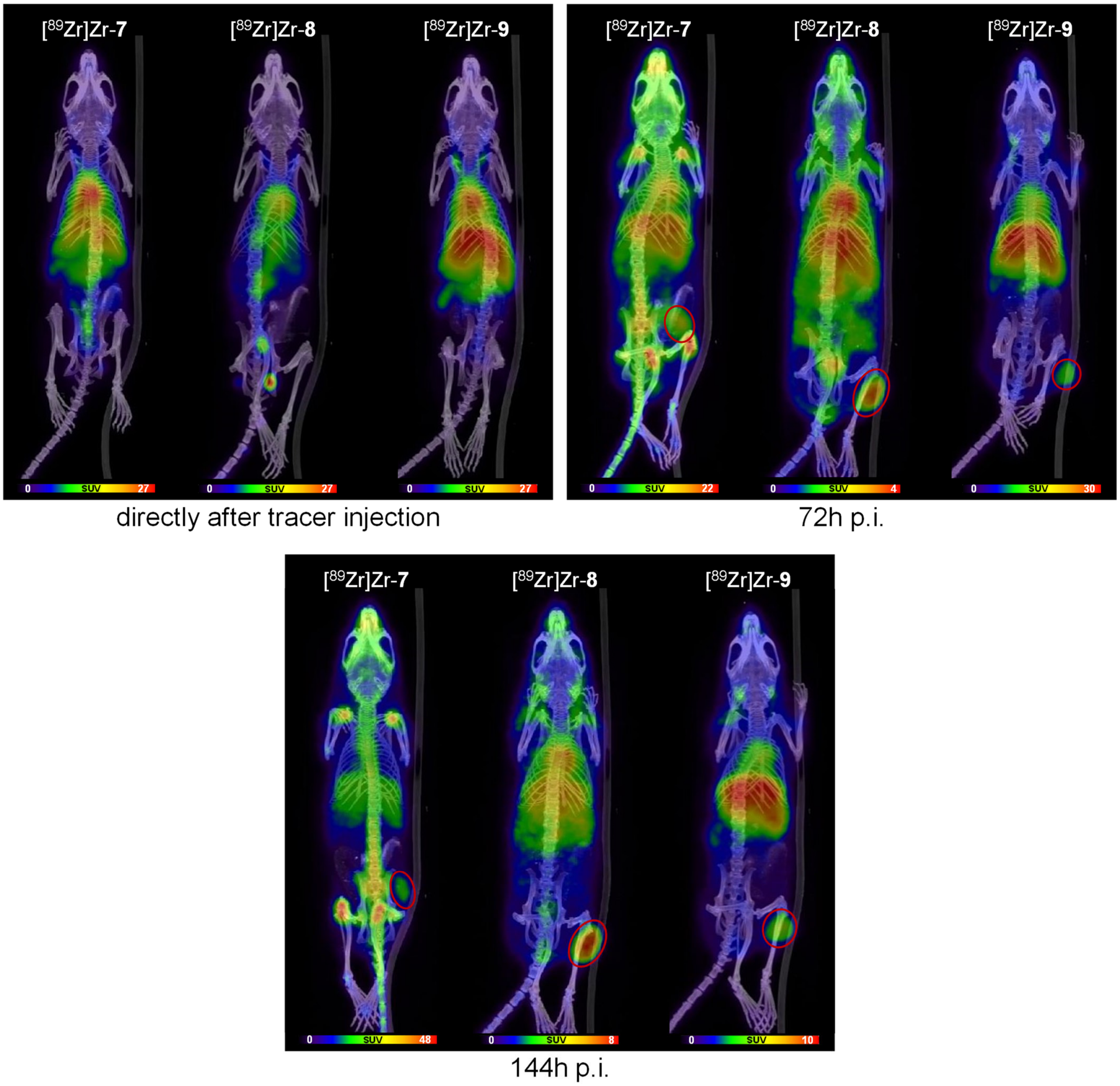

Figure S5: Representative small animal positron emission tomography / computed tomography (PET/CT) images for [^{89}Zr]Zr-DFO-cetuximab ([^{89}Zr]Zr-**7**), [^{89}Zr]Zr-DFO*-cetuximab ([^{89}Zr]Zr-**8**) and [^{89}Zr]Zr-3,4,3-(LI-1,2-HOPO)-cetuximab ([^{89}Zr]Zr-**9**). The images show maximum intensity projections (MIPs) of the whole animals directly after injection (upper panel), at 72h p.i. (middle panel) and 144h p.i. (n = 5 for [^{89}Zr]Zr-**7**, n = 7 for [^{89}Zr]Zr-**8** and n = 7 for [^{89}Zr]Zr-**9**, tumors are encircled).

Table S1: Standardized uptake values obtained from the PET scans for different organs applying [^{89}Zr]Zr-**7**, [^{89}Zr]Zr-**8** or [^{89}Zr]Zr-**9**, normalized on body weight and referenced to brain as reference background tissue for different time points (0h, 72h and 144h) post injection. SUV_{bw} values are given as mean ± standard deviation.

[^{89}Zr]Zr-**7**

	liver	right knee	left knee	heart	tumor
**0 h p.i.**	9.43 ± 1.11	1.27 ± 0.19	1.31 ± 0.20	11.61 ± 0.71	0.86 ± 0.38
**72 h p.i.**	5.14 ± 0.33	6.01 ± 0.55	5.97 ± 0.56	5.56 ± 0.38	4.74 ± 0.71
**144 h p.i.**	4.03 ± 0.62	6.17 ± 0.61	6.03 ± 0.33	3.79 ± 0.37	4.12 ± 0.50

[^{89}Zr]Zr-**8**

	liver	right knee	left knee	heart	tumor
**0 h p.i.**	9.30 ± 0.91	1.24 ± 0.21	1.13 ± 0.15	12.28 ± 0.80	0.51 ± 0.15
**72 h p.i.**	6.06 ± 0.22	2.71 ± 0.38	2.65 ± 0.28	7.55 ± 0.19	4.67 ± 0.95
**144 h p.i.**	6.34 ± 0.60	3.03 ± 0.30	2.74 ± 0.87	7.60 ± 0.41	6.37 ± 2.56

[^{89}Zr]Zr-**9**

	liver	right knee	left knee	heart	tumor
**0 h p.i.**	13.40 ± 2.81	1.47 ± 0.28	1.43 ± 0.20	12.05 ± 0.94	0.67 ± 0.17
**72 h p.i.**	10.02 ± 1.16	3.48 ± 0.52	3.36 ± 0.47	7.45 ± 0.54	6.83 ± 1.47
**144 h p.i.**	10.88 ± 2.07	3.65 ± 0.80	3.46 ± 0.39	7.48 ± 0.56	8.62 ± 2.90
